# Epidural‐Related Maternal Fever Is Associated With Structural Alterations in Vaginal Floral Homeostasis Among Parturients

**DOI:** 10.1155/ogi/7458179

**Published:** 2026-07-27

**Authors:** Chao Xiong, Xuemeng Chen, Wenhu Zhai, Xianjie Zhang

**Affiliations:** ^1^ Department of Anesthesiology, Deyang People’s Hospital, Deyang, Sichuan, China, scu.edu.cn

**Keywords:** ASQ-3, ERMF, labor analgesia, neurodevelopment, vaginal microbiota

## Abstract

**Objective:**

To explore the association between epidural‐related maternal fever (ERMF) and maternal vaginal microbiota, as well as its potential association with early infant neurodevelopment.

**Methods:**

This study enrolled pregnant women who underwent vaginal delivery with epidural analgesia. According to the presence or absence of intrapartum fever (body temperature ≥ 37.5 °C), the participants were divided into either the control group or the fever group. Multiple vaginal swabs were collected from the parturients for further analysis using 16S rRNA amplicon sequencing. The general conditions of both mothers and neonates were recorded. At three months postpartum, infants’ neurodevelopmental status was assessed using the Ages and Stages Questionnaires, Third Edition (ASQ‐3).

**Results:**

Analysis of the vaginal flora demonstrated that Lactobacillus was the dominant genus in both groups. ERMF was not associated with significant changes in the overall alpha diversity of the vaginal microbiota (*p* = 0.32). However, differences were noted in beta diversity (*p* = 0.03). ERMF was associated with an increased relative abundance of Achromobacter and Porphyromonas, and a decreased relative abundance of *Ezakiella* in the vaginal microbiota. In functional prediction analysis, ERMF was associated with decreased expression of protein‐coding genes related to vaginal microbiota stability. Additionally, infants exposed to ERMF exhibited a downward trend in 3‐month ASQ‐3 neurodevelopmental scores, with no statistically significant difference observed (*p* = 0.098). Multivariate logistic regression analysis revealed a statistically significant association between ASQ‐3 scores and duration of labor (OR = 1.189; 95% CI: 1.025 to 1.406; *p* = 0.028).

**Conclusion:**

ERMF is associated with alterations in the structural composition of vaginal microbiota and dysregulation of specific bacterial genera. Labor duration is significantly associated with early infant ASQ‐3 scores. However, this exploratory study has several limitations—including a relatively small sample size and a short follow‐up duration—which preclude definitive conclusions regarding a causal or direct association between ERMF exposure and early infant neurodevelopment. Additional rigorously designed longitudinal studies are warranted to further elucidate and validate this relationship.

**Trial Registration:** Chinese Clinical Trial Registry: ChiCTR2400090531

## 1. Introduction

Epidural analgesia remains the most prevalently used approach for managing labor pain in clinical practice [1]. Nevertheless, epidural analgesia is associated with an elevated risk of intrapartum fever. This phenomenon has been consistently observed in numerous studies and is referred to as “epidural‐related maternal fever” (ERMF) [2, 3]. Approximately 30% of parturients develop a temperature exceeding 37.5°C following epidural analgesia, with the incidence ranging from 10.5% to 36.7% [4]. In women receiving epidural analgesia, elevated maternal temperature can be an independent risk factor for adverse outcomes in full‐term neonates. These adverse outcomes include acidosis, low Apgar scores, and neonatal encephalopathy. Additionally, it increases the likelihood of cesarean delivery [5, 6]. The proportion of neonates experiencing adverse outcomes seems to increase as maternal temperature rises. This poses a serious threat to the safety of both mothers and neonates and also leads to unnecessary medical costs [6, 7].

Evidence from population‐based and animal mechanistic studies suggests that the commensal microbiome is crucial for the early‐life development of both the immune and nervous systems [8]. The composition of the vaginal microbiota and its overall ecological equilibrium are implicated in the pathogenesis of multiple gynecological and endocrine disorders [9]. Exogenous infections, exposure to endocrine‐disrupting chemicals, and other environmental or physiological stressors can perturb hormonal homeostasis, compromise the integrity of the vaginal microenvironment, and thereby induce microbial dysbiosis and reductions in vaginal microbial alpha diversity [10, 11]. Metabolites from the maternal microbiome can influence fetal development, and the establishment of the infant microbiome in the early stages of life is a key determinant of immune system maturation [12]. A healthy colonization of vaginal microbiota is beneficial for the development of microbial function in early infancy [13]. Compared to vaginal delivery, cesarean delivery has been linked to an increased risk of adverse health outcomes in offspring, including neurodevelopmental disorders, immune dysregulation, and metabolic diseases [14, 15]. Although vaginal microbiota transplantation seems to significantly improve infant microbiota homeostasis and early neurological development, its safety and efficacy require further investigation [16]. In conclusion, the homeostasis of the maternal vaginal microbiota is crucial for the establishment of the infant microbiome, immune function, and neurodevelopment during early infancy.

Microbial homeostasis depends on appropriate temperature and microenvironmental conditions [17]. Fever, together with the increase in inflammatory cytokines, may lead to changes in the diversity and functionality of the bacterial flora [18]. Nevertheless, it remains unclear whether ERMF is associated with alterations in the vaginal microbiota, or whether a potential association exists between vaginal microbiota changes and early infant neurodevelopment. To explore these potential associations, we conducted a single‐center prospective case–control study to provide preliminary evidence for the relationship between ERMF, vaginal microbiota alterations, and early infant neurodevelopment.

## 2. Methods

### 2.1. Study Design and Oversight

This study employed a single‐center, single‐blinded, prospective cohort study design. The subjects were recruited from the patient population at Deyang People’s Hospital in Deyang City, Sichuan Province, China. This trial was carried out in accordance with the Declaration of Helsinki.

### 2.2. Participants

From October 2024 to February 2025, this study enrolled primiparas who intended to have a vaginal delivery of a single fetus at Deyang People’s Hospital. Inclusion criteria were as follows: (1) They had no contraindications to epidural anesthesia and voluntarily consented to labor analgesia; (2) the parturients were aged between 18 and 45 years, with the fetus in a cephalic position, carrying a singleton pregnancy at full term, having no contraindications to vaginal delivery, and presenting no obstetric risk factors, such as pregnancy‐induced hypertension syndrome or placenta previa; (3) they had no inflammatory diseases (such as upper respiratory tract infection, urinary tract infection, or reproductive tract infection) and had not experienced fever within 48 h prior to the administration of labor analgesia. In this study, ERMF was defined as an intrapartum temperature ≥ 37.5°C after epidural analgesia, with no clinical or laboratory evidence of acute infection (including negative routine blood tests, no purulent amniotic fluid, and no signs of chorioamnionitis). Infectious fever was strictly excluded.

Exclusions were imposed on women who met any of the following criteria: (1) Confirmed or suspected intrapartum infection, defined as maternal fever exceeding 37.5°C during labor accompanied by clinical signs suggestive of infection; (2) active vaginal infection or administration of systemic antibiotics within 48 h prior to delivery; (3) severe obstetric complications—or other comorbid conditions (e.g., severe psychiatric illness or significant communication impairment)—that contraindicate vaginal delivery; (4) maternal age less than 18 years or greater than 45 years; and (5) established contraindications to epidural analgesia for labor.

Follow‐up was terminated if participants met any of the following criteria: (1) The patient has requested to withdraw from the study and declined to participate in subsequent assessments; (2) the patient has had a cesarean section due to changes in maternal and fetal conditions during labor; and (3) antibiotics have been used for preventive purposes or in cases of suspected infectious fever during labor.

### 2.3. Allocation and Blinding

The primary study objective was to enroll 35 parturients with ERMF. Parturients meeting the inclusion criteria were selected until 35 parturients with ERMF and an uncomplicated vaginal delivery were included in the study. Simultaneously, 35 parturients without ERMF were chosen from the group of parturients who met the inclusion criteria. It was not possible to blind the patients, labor analgesia providers, and midwives to the maternal fever status. Therefore, single blinding was implemented only for the researchers responsible for the 16S rRNA sequencing data analysis and the assessment of the ASQ‐3 scale assessment, who were kept unaware of the group allocation throughout the study.

### 2.4. Study Implementation and Data Collection

Written informed consent for epidural labor analgesia was obtained from all participants prior to the onset of regular uterine contractions and cervical dilation. Vaginal swab samples were collected from the posterior vaginal fornix by trained midwives using sterile swabs and immediately stored at −80°C in a freezer. After the administration of analgesia, the maternal body temperature was continuously monitored. The participants were divided into two groups according to the presence or absence of ERMF (body temperature ≥ 37.5°C), designated as the “fever group” (F group, 35 cases) and the “control group” (C group, 35 cases), respectively. Demographic and pregnancy characteristics of the two groups were recorded, along with the duration of fever in the ERMF group. Before the onset of active labor (before entering the second stage), the midwife recollected the vaginal microbiota swab and stored it in a −80°C freezer for subsequent analysis. Meanwhile, we documented the mother’s delivery process and the postpartum conditions of both the mother and the infant for subsequent analysis. The characteristics of the vaginal flora were identified through 16S rRNA amplicon sequencing (Beijing Nuohezhiyuan Technology Co., Ltd., Beijing, China). The neurodevelopmental level of infants was evaluated using the ASQ‐3 scale in the Department of Child Health Care of our hospital three months postpartum.

### 2.5. 16S rRNA Gene Amplification and Sequencing

Total genomic DNA was isolated from the samples via the magnetic bead method. The V3–V4 hypervariable region of the 16S rRNA gene was amplified using specific primers (515F: 5′‐GTGCCAGCMGCCGCGGTAA‐3′ and 806R: 5′‐GGACTACHVGGGTWTCTAAT‐3′). Library construction was performed using the TruSeq DNA PCR‐Free kit. Eligible libraries were sequenced on the Illumina HiSeq2500 platform, yielding 250‐bp paired‐end reads. After implementing quality control procedures, including data splicing, filtering, and denoising, species annotation was carried out to acquire taxonomic information at the species level along with abundance distribution.

### 2.6. Sample Size Determination

In line with the findings of prior studies and our preliminary data, the mean ASQ‐3 score, which serves to evaluate infant neurodevelopment, was approximately 240 at four months, with a standard deviation of 40 [19]. The results of the pilot study showed that infants born to mothers with ERMF had an average ASQ‐3 score of 210 at three months of age, and the standard deviation was consistent with that of the overall cohort. The sample size ratio of the experimental group to the control group was 1:1. The alpha level for the hypothesis test was set at 0.05, and the beta level was set at 0.2. Considering a 20% loss to follow‐up, a sample size of 70 participants, with 35 in each group, was determined to be necessary. No additional participants were enrolled, as the 35 parturients without ERMF served as the control group.

### 2.7. Statistical Analysis

IBM SPSS Statistics 25.0 (IBM Corp., Armonk, NY, USA) and R software (Version 4.1.0) were used for data management and statistical analysis. The Kolmogorov–Smirnov test was used to evaluate the normality of the data distribution. Data with a normal distribution were presented as the mean ± standard deviation, whereas non‐normally distributed data were summarized using medians and interquartile ranges. For comparisons among multiple groups, one‐way ANOVA was employed; for pairwise comparisons, either the *t*‐test or Wilcoxon rank‐sum test was utilized, depending on the data distribution. Binary variables were reported as frequencies and percentages and analyzed using the chi‐square test or Fisher’s exact test, as appropriate. QIIME2 software was used for the biological analysis of vaginal microbiota to calculate *α*‐diversity and *β*‐diversity indices. Data visualization and interpretation were carried out using R software. Group‐wise statistical comparisons were performed using the Kruskal–Wallis test, followed by Tukey’s post hoc test. A comprehensive set of statistical methods, including ANOSIM, ADONIS, *t*‐tests, MetagenomeSeq, and LEfSe, was used to identify taxa with differential abundance. Statistical significance was defined as a two‐tailed *p* value < 0.05.

Multivariable logistic regression was employed to assess the associations between prespecified risk factors and ASQ‐3‐based neurodevelopmental outcomes in infants. The outcome variable was dichotomized as “normal development” versus “at risk for developmental delay,” defined using a cutoff of 1 standard deviation below the population mean on the ASQ‐3 total score. Key predictors included maternal exposure to elevated maternal rectal temperature during labor (fever group vs. control group), maternal age, gestational age at birth, presence of maternal comorbidities, and duration of labor. Covariates were selected a priori based on robust empirical evidence linking them to infant neurodevelopmental outcomes, as documented in the existing literature. Results are reported as odds ratios (ORs) with corresponding 95% confidence intervals (CIs). Model calibration was evaluated using the Hosmer–Lemeshow test; a nonsignificant chi‐square statistic (*p* ≥ 0.05) indicated adequate goodness of fit. Statistical significance was defined as two‐sided *p* < 0.05.

## 3. Results

### 3.1. Overview of the Perinatal Health Status of Mothers and Newborns

**FIGURE 1:**
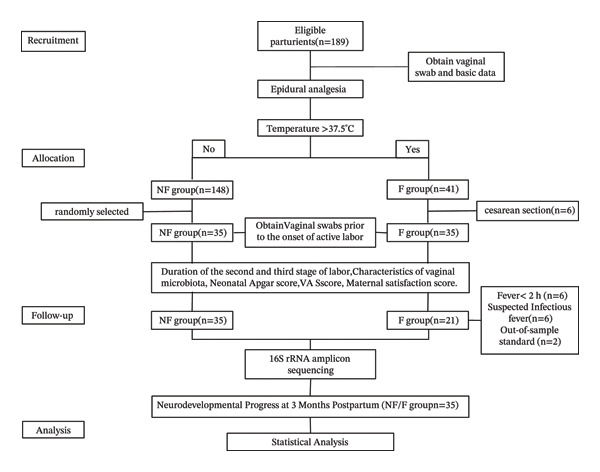
Consolidated standards of reporting trials (CONSORT) flow diagram for the study protocol.

From October 2024 to February 2025, a total of 189 primiparous women who met the inclusion criteria and intended to have a vaginal delivery of a singleton pregnancy were recruited at Deyang People’s Hospital. All participants received epidural labor analgesia, with an overall ERMF incidence of 21.7% in the study cohort. The study flowchart is shown in Figure 1. Analysis of maternal and fetal prenatal characteristics revealed no statistically significant differences between groups. Postpartum assessment of maternal and neonatal outcomes indicated that the Apgar score at 1 min was lower in the F group compared with the C group (*p* = 0.099), though this difference did not reach statistical significance. No other intergroup differences reached statistical significance (Table 1).

**TABLE 1:** Comparison of maternal and infant baseline characteristics between the F and the C groups.

Maternal and infant characteristics	F group (*n* = 21)	C group (*n* = 35)	Statistic	*p* value
Age (years), mean ± SD	28.54 ± 3.93	28.00 ± 2.43	0.655	0.515
BMI (kg/m^2^), mean ± SD	26.19 ± 2.04	27.26 ± 2.67	1.690	0.097
Gestational age (weeks), median (25%, 75%)	39 (39, 40)	40 (39, 40)	0.996	0.324
Complication frequency (%)			—	0.412
No	14 (66.67%)	19 (54.29%)		
Yes	7 (33.33%)	16 (45.71%)		
Cervical orifice size (cm), median (25%, 75%)	3 (2.5, 3)	3 (2.5, 3)	0.693	0.491
VAS score before analgesia, mean ± SD	9.143 ± 0.66	8.87 ± 0.58	1.526	0.133
Puncture interval, frequency (%)			—	0.375
L2‐3	1 (4.76%)	0 (0%)		
L3‐4	20 (95.24%)	35 (100%)		
VAS score after analgesia, mean ± SD	2.33 ± 0.73	2.27 ± 0.667	0.249	0.804
First stage of labor (h), median (25%, 75%)	12.20 (10.00, 13.75)	10.00 (8.30, 15.4)	0.485	0.6294
Second stage of labor (h), median (25%, 75%)	1.7 (1.10, 2.05)	1.6 (1.20, 2.40)	1.057	0.295
Third stage of labor (min), median (25%, 75%)	6 (5, 7)	5 (4, 7)	0.989	0.327
Apgar score at 1 min, mean ± SD	9.29 ± 0.64	9.60 ± 0.69	1.684	0.099
Apgar score at 5 min, mean ± SD	9.95 ± 0.22	9.91 ± 0.28	0.528	0.599
Apgar score at 10 min, mean ± SD	10	10	—	—
Birth weight (g), mean ± SD	3242 ± 232.41	3239 ± 284.26	0.040	0.968
Birth height (cm), median (25%, 75%)	51 (50, 51)	50 (50, 51)	0.111	0.912
Hemorrhage (mL), mean ± SD	260 ± 70.14	307 ± 133.15	1.494	0.141
Fever characteristics				
Duration of fever (h), mean ± SD	5.18 ± 2.16			
Degree of fever, mean ± SD	37.99 ± 0.40			
Pain management satisfaction, mean ± SD	9.38 ± 0.50	9.2 ± 0.58	1.184	0.242
Mean VAS score, mean ± SD	2.71 ± 0.77	2.70 ± 0.64	0.071	0.943
ASQ‐3, mean ± SD	225 ± 10.12	230 ± 11.11	1.684	0.098
Number of vaginal examinations, mean ± SD	3.52 ± 1.03	3.20 ± 1.05	1.124	0.266

### 3.2. Characteristics of the Species Abundance and Diversity of Vaginal Microbiota in Parturient Women

Vaginal swabs were obtained before labor analgesia and prior to the start of the second stage of labor. In the C group, the vaginal swabs were classified into pre‐analgesia (PC) and post‐analgesia (AC) samples. For the F group, the vaginal flora specimens were likewise divided into pre‐analgesia (PF) and post‐analgesia (AF) groups. Among the fever group, six parturients had transient fevers lasting less than two hours, six presented symptoms of infectious fever that necessitated antibiotic treatment, and two provided noncompliant specimens. To improve the experimental rigor, these unqualified or ambiguous samples were excluded from subsequent analysis.

**FIGURE 2:**
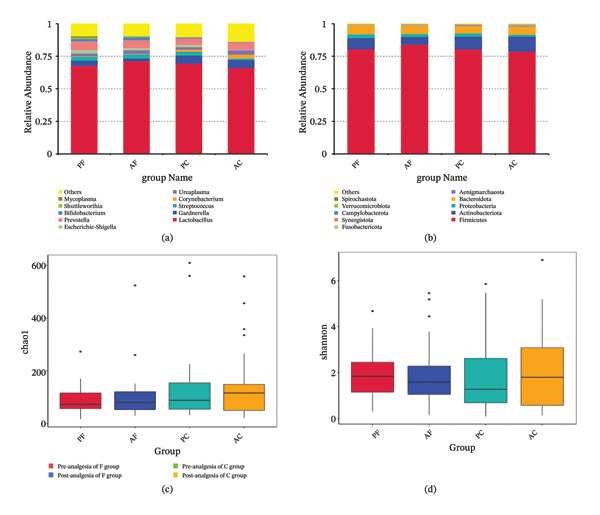
Analysis of species distribution and diversity in the vaginal microbiota. (a) Species abundance at the genus level. (b) Species abundance at the phylum level. (c) Alpha diversity analysis based on the Chao index. (d) Alpha diversity analysis based on the Shannon index.

Based on the annotation results of species at different taxonomic levels, the top 10 species with the highest relative abundances at each taxonomic level (phylum and genus) were determined. The remaining species were grouped as “Others” to create bar charts presenting the relative abundances of species annotation results at various taxonomic levels for each group (Figure 2). Lactobacillus (Firmicutes) was identified as the dominant genus in the vaginal microbiota, and the structural composition of genera (phyla) across all groups remained consistent. Analysis of microbial diversity revealed no statistically significant differences in alpha diversity among all groups (all *p* > 0.05).

### 3.3. ERMF Was Associated With Alterations in the Beta Diversity of Vaginal Microbiota and Increased Relative Abundance of Opportunistic Pathogenic Bacteria

**FIGURE 3:**
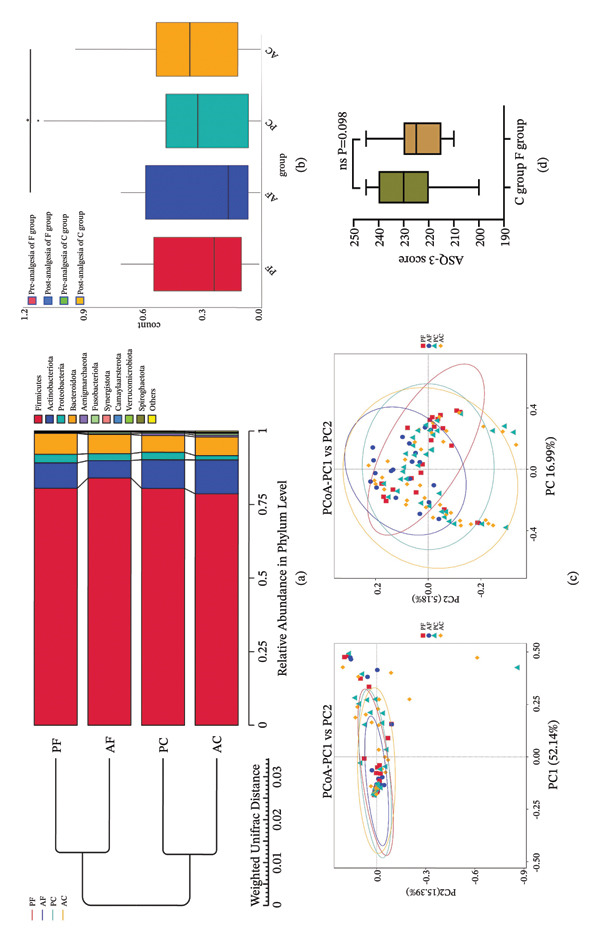
Comprehensive analysis of beta diversity in microbiota: (a) UPGMA clustering analysis was performed based on the weighted UniFrac distance matrix at the phylum level. (b) Intergroup diversity analysis using the weighted UniFrac Tukey HSD test, ^∗^ *p* < 0.05. (c) Two‐dimensional principal coordinates analysis (PCoA) plots are presented, with the left panel based on the weighted UniFrac distance metric and the right panel based on the unweighted UniFrac distance metric. (d) ASQ‐3 scores of infants in Group C and Group F at 3 months of age.

**FIGURE 4:**
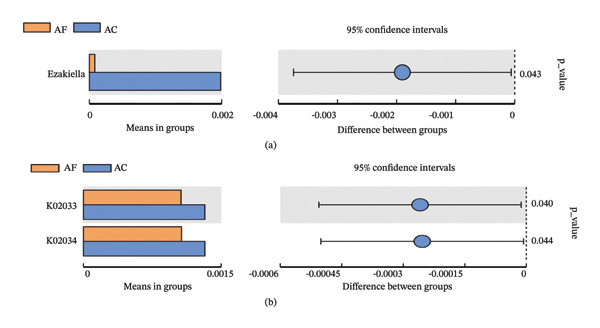
Differential species analysis and functional prediction. (a) Species exhibiting intergroup differential abundance according to classification levels. (b) Functional distinctions among different groups as determined by the Kyoto Encyclopedia of Genes and Genomes (KEGG) database.

In the analysis of the beta diversity of vaginal microbiota species across different groups, a significant difference was detected between the AC group and the AF group (*p* = 0.03) through Tukey’s HSD variance analysis of weighted UniFrac among multiple groups (Figure 3). To delve deeper into the differentially abundant species between the groups, a *t*‐test was carried out to identify significant differences at each taxonomic classification level. A remarkable difference in the *Ezakiella* genus was found between the AC and AF groups. Moreover, by analyzing species abundance at various taxonomic levels, the MetagenomeSeq method was utilized to conduct hypothesis testing on the intergroup species abundance data. In the ERMF group, the relative abundances of Porphyromonas and Achromobacter were significantly increased compared with the control group. These genera are opportunistic pathogens, and their increased abundance has been linked to vaginal microecological imbalance and inflammatory status in previous studies. In the functional prediction analysis using PICRUSt2, the expression levels of K02033 and K02034 genes were higher in the AC group compared to the AF group (Figure 4).

### 3.4. Infants Exposed to ERMF Showed a Downward Trend in 3‐Month Neurodevelopmental Scores With No Statistically Significant Difference

To assess the potential effect of ERMF on early infant neurodevelopment, the ASQ‐3 was used to evaluate neurodevelopment in 3‐month‐old infants. This instrument assesses five domains: communication, gross motor skills, fine motor skills, problem‐solving, and personal–social development. Our results showed that infants in the intrapartum fever group tended to have lower ASQ‐3 scores compared to those in the control group. Nevertheless, this downward trend in the fever group did not reach statistical significance compared with the control group (225 ± 10.12 vs 230 ± 11.11, *p* = 0.098, no statistical significance) (Table 1 and Figure 3d). These findings are exploratory and should be interpreted with caution given the limited sample size (*n* = 21 in the fever group after exclusions) and the lack of full multivariable adjustment for this secondary neurodevelopmental outcome.

### 3.5. Infant Neurodevelopmental Outcomes at 3 Months Are Significantly Associated With Maternal Duration of Labor

To control for potential confounding effects of maternal age, duration of labor, and the number of vaginal examinations on infant early neurodevelopmental outcomes, multivariate logistic regression was employed with the dichotomized ASQ‐3 outcome (normal vs. at risk for developmental delay) as the dependent variable.

Among the 56 infants who completed the 3‐month ASQ‐3 assessment, 16 infants (28.57%) were classified as at risk for developmental delay based on the 1 SD cutoff criterion. The analysis revealed a statistically significant association between longer duration of labor and increased odds of at‐risk neurodevelopmental status in infants (OR = 1.189; 95% CI: 1.025 to 1.406; *p* = 0.028). In contrast, no statistically robust evidence was found to support an association between ERMF exposure and impaired early neurodevelopment (OR = 1.790; 95% CI: 0.477 to 6.968; *p* = 0.387) (Table 2). The Hosmer–Lemeshow goodness‐of‐fit test produced a *p* value of 0.448, suggesting that the model fits the data adequately. However, given the limited sample size and consequent constraints on statistical power, the findings should be interpreted with appropriate caution.

**TABLE 2:** Multivariate logistic regression analysis of ASQ‐3 score.

Dependent variable	Independent variable	Standard error	*Z*	*p*	OR	95% CI
ASQ‐3	Constant	13.560	0.92	0.356	—	—
	Age	0.130	0.53	0.595	1.069	0.838 to 1.383
	Duration of labor	0.079	2.20	0.028^∗^	1.189	1.025 to 1.406
	ERMF	0.674	0.86	0.387	1.790	0.477 to 6.968
	Number of vaginal examinations	0.333	1.30	0.192	0.648	0.324 to 1.223
	Complications	0.756	0.57	0.569	0.650	0.138 to 2.826
	Gestational age	0.359	0.62	0.537	1.248	0.636 to 2.734

^∗^Statistical significance at the *p* < 0.05 level.

## 4. Discussion

ERMF is a major concern for both anesthesiologists and obstetricians. The adverse impacts of ERMF on maternal and neonatal health are wide‐ranging and long‐term. Nevertheless, the mechanisms through which this fever increases the risk of adverse neonatal outcomes remain unclear [20, 21]. This preliminary exploratory study found that ERMF exposure was associated with structural changes of maternal vaginal microbiota and potential reduction of its stability. Labor duration is significantly associated with early infant ASQ‐3 scores, while, given the limitations of the study, no clear association could be established with ERMF.

All microorganisms have an optimal range of temperature, humidity, and microenvironmental conditions that promote their growth and reproduction [17]. The research also showed a mutual regulatory relationship between inflammatory cytokines and the vaginal microbiota. An increase in inflammatory cytokines can lead to an imbalance of the vaginal flora [18, 22]. Therefore, ERMF may potentially alter vaginal microbiota stability by modulating maternal body temperature and inflammatory cytokine levels. We collected vaginal swabs at various time points before and after the administration of epidural labor analgesia, stratified according to the presence or absence of fever, and performed 16S rRNA amplicon sequencing. The findings indicated that ERMF did not seem to significantly change the alpha diversity of the vaginal microbiota across multiple groups. Nevertheless, after the initiation of labor analgesia, a difference in beta diversity, as measured by weighted UniFrac distance, was noted between the control group and the fever group. These results seem reasonable, and the reasons are diverse. First, the severity and duration of fever appear to have a relatively limited impact on the alpha diversity of the vaginal microbiota. Second, during the labor waiting period, midwives often perform vaginal examinations and artificially rupture the amniotic sac, which results in amniotic fluid flushing the vagina. Compared to elevated inflammatory markers and exposure to environmental microbial flora, these factors seem to have a more significant impact on the structure of the vaginal microbiota. In the diversity analysis, it was noted that the within‐group variability of the samples was greater than the between‐group differences. Certain differential species were identified at the species classification level. Specifically, compared with the prefever conditions, the abundance of Porphyromonas and Achromobacter increased in the vaginal microbiota of parturients after fever. These taxa are generally opportunistic pathogens in the vaginal microbiota, and their overgrowth is regarded as one of the indicators of vaginal microecological imbalance. The protease activity of Porphyromonas vaginalis impairs coagulation and the extracellular matrix within the cervicovaginal niche. This impairment is closely associated with cervical cancer, bacterial vaginosis, and other gynecological inflammatory conditions [23, 24]. *Achromobacterium* is involved in obstetric and gynecological disorders, such as premature rupture of membranes and vulvar pain syndrome [25, 26]. Thus, ERMF is associated with alterations in the vaginal microenvironment, which may contribute to vaginal microbial dysbiosis in parturients.

Notably, after labor analgesia, distinct differences in species composition are evident between the vaginal microbiota of the fever group and that of the control group. Specifically, the genus *Ezakiella* was found to be significantly more abundant in the control group. The *Ezakiella* genus, a bacterial genus discovered in 2017, was initially identified as part of the intestinal microbiota [27]. Due to the anatomical proximity between the anus and the vagina, this bacterial genus was subsequently detected in the vagina, with a higher prevalence noted prepubertally [28]. Nevertheless, its exact pathophysiological role remains to be comprehensively elucidated. Several studies have shown its close association with inflammatory bowel disease [29], chlamydia‐associated vaginitis [30], relapsing–remitting multiple sclerosis [31], and its significant involvement in the microbial environment of the urinary system [32]. Due to the low prevalence of ERMF, the numerous associated risk factors, and the potential for cross‐contamination among the urethra, vagina, and anus caused by repeated vaginal examinations, it is not possible to establish a direct association or causal relationship between *Ezakiella* and ERMF in this study, due to the limited sample size, potential confounding factors (repeated vaginal examinations and anatomical cross‐contamination), and insufficient functional verification. Further functional analysis seems to be necessary.

Subsequently, a functional prediction analysis was carried out. Based on the KEGG database, significant disparities in functional gene expression were detected between the fever group and the control group. A search in the KEGG database revealed that these genes encode peptide/nickel transport system permease proteins. The transport of peptides and nickel is of great significance in enabling microorganisms to adapt to environmental challenges, such as nutrient deficiency and host immune pressure [33, 34]. Therefore, we hypothesize that, compared with the fever group, the vaginal microbiota in the control group may possess a stronger ability to adapt to the environment. This finding is consistent with the aforementioned results, which indicate that ERMF is associated with changes in the vaginal microbiota microenvironment, potentially reducing the stability of the vaginal microbiota.

In addition to examining the impact of ERMF on the vaginal microbiota, an evaluation of the early neurodevelopmental status of infants was also carried out. Based on previous research findings, intrapartum fever is closely associated with adverse neonatal outcomes, thus increasing the risk of neurological disorders in newborns [7, 20]. Nevertheless, most of the existing studies rely on retrospective cohort designs. Due to their inherent limitations, these designs prevent causal inference, leading to a lack of solid evidence [35]. In this research, the ASQ‐3 assessment tool was utilized to evaluate the neurodevelopmental scores of 3‐month‐old infants. A downward trend in ASQ‐3 scores was noted among infants in the fever group. These results only showed a nonstatistically significant downward trend in neurodevelopmental scores and cannot indicate a direct influence of ERMF on infant neurodevelopment. Multivariate logistic regression analysis, after adjusting for confounding factors, revealed that the duration of labor was associated with early neurodevelopment in infants. The potential association needs to be verified by further large‐sample studies with long‐term follow‐up. Nevertheless, the exact underlying mechanisms require further exploration. In a noninfectious fever model of pregnant rats, it was found that fever during parturition could lead to the activation of microglia and an increase in inflammatory cytokines in the brains of neonatal rats, which may potentially affect the neurodevelopment of the offspring [36]. Simultaneously, the analysis indicated that ERMF seems to be linked to a lower Apgar score in the first minute after birth. Thus, the combined effects of fever itself, elevated inflammatory cytokines, and the slight downward trend of 1‐min Apgar score may be potential factors related to the downward trend of neurodevelopmental scores in infants, but no causal or mediating effect was confirmed in this study. The associations between these factors and the incidence of ERMF, along with its impact on fetal outcomes, merit further research.

This study has several important limitations that need to be clarified:

1. Limited sample size and reduced statistical power: This was a single‐center study with an initial sample size of 70 participants (35 per group). However, 14 samples in the ERMF group were excluded due to transient fever, suspected infectious fever, and noncompliant specimens, resulting in a reduced effective sample size (*n* = 21 in the fever group) and diminished statistical power—particularly for the secondary outcome of early infant neurodevelopment. Consequently, these findings are exploratory and hypothesis‐generating, with limited generalizability to other cohorts.
2. Lack of full multivariable adjustment for microbiome analyses: We did not perform comprehensive multivariate analysis to control for key confounding factors that may affect vaginal microbiota composition, including the frequency of vaginal examinations during labor, duration of membrane rupture, vaginal inflammatory marker levels, and maternal gestational metabolic status. This limitation should be considered when interpreting the microbiome findings, as it may lead to residual confounding bias.
3. Unestablished direct association between microbiota and neurodevelopment: Given the paucity of statistically significant findings, we did not extend infant follow‐up beyond three months. Furthermore, we did not assess the direct association between alterations in vaginal microbiota composition and infant neurodevelopmental outcomes. At this stage, it is premature to infer either an association or a causal relationship among ERMF exposure, vaginal microbiota shifts, and infant neurodevelopment.
4. Lack of key follow‐up data: Fecal samples (for intestinal microbiota analysis) were not collected from infants, which represents a critical gap in investigating maternal vaginal microbiota vertical transmission and its potential impact on the infant gut–brain axis. Future research will address these gaps through large‐sample multicenter prospective studies, animal models with strict variable control, and long‐term infant follow‐up.

## 5. Conclusion

This single‐center prospective cohort study provides preliminary evidence that ERMF is associated with structural alterations in vaginal microbiota, increased relative abundances of opportunistic pathogens, and potential reductions in vaginal microbiota stability among parturients. Regarding the secondary outcome of infant early neurodevelopment, the ERMF group exhibited a nonstatistically significant downward trend in 3‐month ASQ‐3 scores; this trend was associated with longer duration of labor. However, no statistically robust evidence was found to support an association between ERMF exposure and impaired early neurodevelopment. This exploratory, hypothesis‐generating study has several important limitations, including the reduced sample size, the exploratory nature of the analyses, and the lack of full multivariable adjustment for microbiome findings. The findings regarding infant neurodevelopment should be interpreted with caution. The potential causal relationship between ERMF, vaginal microbiota alterations, and infant long‐term neurodevelopmental outcomes needs to be further verified by large‐sample, multicenter prospective studies with strict confounding control and long‐term infant follow‐up.

**Nomenclature**
ERMF: Epidural‐related maternal fever
ASQ‐3: Ages and Stages Questionnaire‐3

## Author Contributions

Chao Xiong and Xianjie Zhang conceived and designed the study. Wenhu Zhai and Xuemeng Chen were responsible for collecting case data. Xuemeng Chen drafted the manuscript. Chao Xiong completed the final revisions of the manuscript.

## Funding

This study was supported by the Xinglin Scholar Special Project of Chengdu University of Traditional Chinese Medicine, Project Supporting Fund No. XJ2023005701.

## Disclosure

All authors revised the manuscript and gave the final approval of the version to be submitted.

## Ethics Statement

The study protocol has been approved by the Ethics Committee of Deyang People’s Hospital, Deyang City, Sichuan Province, China (2024–04‐064‐K01), and all methods were performed in accordance with the relevant guidelines and regulations.

## Consent

Consent for publication was obtained from all individual participants included in the study.

## Conflicts of Interest

The authors declare no conflicts of interest.

## Data Availability

The majority of data are included in the manuscript. The microbiome analysis data are available upon request by contacting the corresponding author via email.
